# The compounding impact of the social determinants of health and COVID-19 on the mental health of young workers in Canada during the COVID-19 pandemic: A qualitative, arts-based study

**DOI:** 10.1371/journal.pone.0309720

**Published:** 2024-08-29

**Authors:** Roberta L. Woodgate, Corinne A. Isaak, Julia Witt, Pauline Tennent, Ashley Bell

**Affiliations:** 1 College of Nursing, Rady Faculty of Health Sciences, University of Manitoba, Winnipeg, Manitoba, Canada; 2 Department of Economics, Faculty of Arts, University of Manitoba, Winnipeg, Manitoba, Canada; 3 Centre for Human Rights Research, Faculty of Law, University of Manitoba, Winnipeg, Manitoba, Canada; St John’s University, UNITED STATES OF AMERICA

## Abstract

The COVID-19 pandemic, a global health crisis, was acutely felt in the labour market for many young workers. Importantly, precarious employment, identified as an emergent social determinant of health, may negatively affect the mental health and well-being of young workers. To this end, we engaged with young workers to understand their workplace needs and challenges in the COVID-19 era and hear their recommendations for action. Semi-structured interviews and a graphic recording focus group were conducted with 33 young workers aged 18–26 years old in Manitoba, Canada, who had worked a minimum of 30 hours per week prior to COVID-19 onset and were living independent of their parents. Analysis involved delineating units of meaning from the data, clustering these to form thematic statements and extracting themes. Second-level analysis involved applying themes and sub-themes to a social determinants of health framework. The multifaceted, compounding realities of young workers’ pre-COVID-19 employment situations were amplified by the COVID-19 pandemic, adversely impacting young worker’s mental health. Unique findings from this study highlight the generational differences in this cohort, who are opposed to participating in fragmented systemic structures (neoliberalism) and inequitable employment conditions, and who yearn for social inclusion and work-life balance. Their recommendations for government and employers call for permanent and stable employment opportunities, economic and mental health supports, and space to be heard and valued, as they navigate the many life course challenges as emerging adults. Societies are dependent on young workers to develop and support the Canadian economy for future generations. Thus, it is a critical that recommendations proposed by young workers in this study be acted upon and implemented to provide an equitable, stable, and supportive future for young workers in Canada and beyond.

## Introduction

The COVID-19 pandemic has been characterised as a co-occurring global health and economic crisis [1], a mental health (MH) crisis [2], a labour market crisis, ‘a crisis of precarious work itself’ (p. 345) [3], and ‘a pandemic of inequality’ (p. 1) [4]. Young people, often in precarious employment positions (i.e. cashiers, delivery drivers), were among those risking their well-being to deliver essential services during the COVID-19 pandemic. Many of these young earners are in working poverty, with limited financial resources, choice, social protections, and often have an inability to fully advocate for their rights [5]. COVID-19 has served to further intensify the precarity of many young workers (YW), and youth are among those at greater risk of being emotionally and psychologically affected by the pandemic [6–8], compounded by pre-existing MH challenges in Canada [9, 10]. While there have been efforts to address these needs (i.e., emergency benefits), information from Canadian YW about their employment experiences during the pandemic and the resulting impacts on their MH is lacking.

### Youth employment

The labour market for young people today looks markedly different than it did a generation ago, with greater uncertainty and instability present for YW [11, 12] as well as higher rates of unemployment and underemployment [11, 13]. Canadian youth have long expressed concerns regarding the number and quality of jobs available to them [14]. Many are employed in part-time and/or contract work [11], and a substantial number of young people who completed post-secondary schooling are not employed in a position that requires their degree [15]. For young people who are unemployed, they experience greater barriers and challenges in (re)entering the workforce, as compared to more established workers [16]. Furthermore, discrimination and racism are significant barriers to employment and advancement in the Canadian labour market [17], particularly for newcomer, BIPOC (Black, Indigenous, and People of Colour), and 2SLGBTQIA+ youth who often experience these factors during hiring [18] and throughout their employment, which present barriers to safe, secure, and stable employment [19].

Contributing to the current status of youth employment, neoliberalism, a dominant ideology in Western societies [20], has shaped the employment market young people are involved in [20, 21]. Neoliberalism is a set of social, economic, ideological and political practices, characterized by individualism, competition, and an emphasis on the benefits of free trade and flexible labour [20, 21]. As such, this has resulted in a rise in precarious employment, which are often positions held by young people [20, 21]. While no global agreement on the definition of precarious employment exists [22], the International Labour Organization (ILO) defines precarious employment using terms such as “uncertainty”, “ambiguous”, “lack of benefits” and “low pay”.(p. 30) [23] Some examples of precarious employment include short-term contract work, staff-for-hire (“gig”) work, and involuntary part-time work. Of concern, precarious employment is increasingly common amongst YW with all levels of education. Not surprisingly, Statistics Canada has reported that youth aged 15–24 are more likely than other age groups to feel insecure about their continued employment, with 31% of youth reporting this as having a major impact on their ability to make ends meet [24]. These trends are worrisome given society’s dependence on YW to develop and support future economies [12]. Importantly, precarious employment has been identified as an emergent social determinant of health [25] and is linked to inequity and poverty [20]. It is also a risk factor that may negatively affect the MH and well-being of YW in these positions [26–29]. It also impacts YW life course, as many YW in precarious employment find it difficult to plan for the future and experience a reduced sense of autonomy, and, as such, find themselves delaying key milestones of adulthood, such as marriage [30, 31]. In addition to affecting the individual YW, precarious employment also affects communities, constituting a public health issue [32]. This has the potential for societal burdens and economic consequences [33], intergenerational poverty [33] and an increased risk for long-term social and economic exclusion and disengagement [34]. In attempts to manage the precarity of their existing employment, YW find themselves turning to the connection of others and interdependence in their work [11]. As such, some YW of this generation are turning away from this neoliberal ideal of employment, through their recognition that precarious work involves perpetuating uneven power relations [21].

### COVID-19 impact on young workers

COVID-19 has had a profound impact on YW, including implications for both their employment and their MH. YW, a group with particularly vulnerable employment status, have been especially susceptible to COVID-19-related changes in the labour market [13], including challenges in finding and gaining stable employment, a key feature of their life stage [35]. Young people are also notably susceptible to economic shocks as a result of being newer to the labour market [16]. They often work in jobs with less employment protection and are typically the first to be fired when workplaces restructure [16]. Previous experiences with economic recessions have noted greater losses in employment and higher instances of unemployment among young people, both in the initial short-term period, as well as long-term [16]. The implications of this being that it often takes longer for the labour market of young people to rebound, with the negative impacts persisting for years [16]. Furthermore, these impacts can also worsen existing inequities among YW [13].

COVID-19 has had a disproportionate impact on the unemployment rates of YW, compared with older workers [36–39]. Many young people lost their jobs or had a reduction in the number of hours they worked [35, 40], which was amplified for equity deserving people [16, 35]. Industries that typically employ young people, including retail and service sectors, were subject to pandemic closures and consequent job loss for YW [35]. As well, young people often hold less secure (precarious) positions, which may contribute to the larger decline of employment among YW [39]. As well, for those in precarious employment positions, COVID-19 had an amplified effect on them, as they had limited access to income, social, and employment protections, such as paid sick time or paid leave [13]. These employment impacts also had the greatest effect on equity deserving YW [3, 41]. For instance, loss of work hours during COVID-19 was more common among Canadian YW who identified as Indigenous (45%) or who lived with a disability (34%) [42]. Young people who were unemployed during the pandemic will likely experience long-term disadvantages competing in the existing labour market [35]. They have also noted greater difficulties entering the workforce during the pandemic [40]. This has limited job opportunities and career development for young people [43]. In turn, this is also expected to disrupt key transitions into adulthood that rely on financial stability, such as parenthood and homeownership [35].

With mental illness an existing issue amongst YW in Canada pre-pandemic [44], COVID-19 has seen further declines in the MH of YW [2]. On the whole, the COVID-19 pandemic has worsened the MH of young people in Canada, and exacerbated pre-existing MH disparities [45]. The pandemic has seen more young people experiencing symptoms of depression, anxiety, stress, and worry, as well as an increase in the severity of these symptoms [46–49]., and an increase in their use of substances (such as alcohol and cannabis) [48]. These MH concerns were amplified for racialized young Canadians [50], as well as those with financial worry, lower income people, and people who were laid off/not working during the pandemic [47, 48].

Beyond this, people who worked in essential and frontline positions, which includes a larger portion of YW, were also at increased risk of exposure to and contracting COVID-19 [36, 51]. This was further amplified for BIPOC individuals who tend to have higher rates of employment in these sectors [36]. Essential and frontline workers further note a higher instance of MH challenges [52–54], and had greater stress and worry associated with contracting COVID-19, passing it on to people close to them, and accessing proper personal protective equipment [52, 53–56].

### Engaging young workers

In supporting our YW, we need to actively engage them in discussions and processes aimed at understanding their employment experiences. To create a more sustainable workforce following the COVID-19 pandemic, it is critical to involve YW in an open discussion to develop solutions resulting from the labour crisis of the pandemic [13]. However, limited qualitative research presently exists on Canadian YW’ experiences during this historical period. To this end, this study aligns with the ILO’s recommendation to engage in social dialogue with employees to address pandemic-influenced labour market challenges [57]. This research provides a unique contribution to the literature to understand the impacts of the COVID-19 pandemic on the mental health of young workers within the Canadian context, through exploring topics impacting their employment experiences, finances, physical health, support and social networks.

Regarding youth as experts to empower their research involvement [58, 59], we engaged with YW to detail their employment challenges in the COVID-19 era and to identify solutions that can result in more supportive, responsive, safe, and healthy workplaces and improved health outcomes. Specifically, the research questions are:

1. What are the workplace experiences and challenges of young workers in the COVID-19 era?
2. What are young workers’ solutions to identified challenges that align with the needs and priorities of young people?

## Methods

### Study context and design

This qualitative, arts-based study took place in Manitoba (MB), a central Canadian province from October 2020 to April 2022, during various phases of the COVID-19 pandemic. As in other jurisdictions, Manitoba experienced four pandemic waves between March 2020 through March 2022 including an Omicron wave (4^th^). The first COVID-19 pandemic lockdown in Manitoba was declared on April 1, 2020 [60]. On March 15, 2022 all Manitoba public health restrictions were lifted [61]. During this time period the Manitoba Government was led by the Progressive Conservatives (PCs), and similar to other regions in Canada, Manitoba was faced with significant economic, public health, and social challenges resulting from the COVID-19 pandemic [62]. During this time period, the unemployment rate in Manitoba was 5.0% in December 2019 [63], 5.5% in October 2021, and 4.6% in October 2022 [64].

Multiple data collection methods were used to examine young workers’ perspectives, providing complementary insights that may otherwise be difficult to access if using a single method [65, 66]. Specifically, semi-structured interviews followed by a graphic recording focus group were used to provide a creative way for study participants to share their stories and illuminate the human dimensions of health and employment experiences [67, 68]. Graphic recording, an arts-based data collection method was employed to listen for key ideas and visually document them (i.e., illustrations, text), enabling youth to visualize their words and conversation patterns in real time [69]. This method was selected as it provides opportunity for young workers to make sense of their work collectively, and to link graphic recordings to the corresponding interview data, creating visual representations of the text-based findings and enhancing understanding of YW experiences with COVID-19. Open-ended guides were created for interviews and focus groups in collaboration with our community partner, focusing on YW’s employment experiences during COVID-19, economic, social, and MH challenges, and recommendations for employers and policymakers to support YW and improve their employment situations. This qualitative study followed the Consolidates Criteria for Reporting Qualitative Research (COREQ) [70]. The principal investigator (PI) leads a well-established impactful research program and team in the study of child/youth well-being with expertise in qualitative research including youth-centered and arts-based methods. Engagement with young workers was facilitated via the long-standing research relationship the PI has with the community partner, NorWest Co-op Community Health [71], and collaborating organization Youth Employment Services Manitoba (YES Manitoba), a long-term partner to NorWest. Both organizations are situated in the downtown or central areas of a major city in Manitoba. To ensure that all participants view their participation positively and feel a sense of emotional safety throughout the research process, we adopted the qualities identified in Woodgate’s framework “sustaining mindful presence.”[59]

### Participant recruitment and data collection

Participant recruitment and data collection took place between October 20, 2020 and April 25, 2022. Using a combination of purposive and snowball sampling, young workers (YW) aged 18–26 years, who had worked a minimum of 30 hours per week prior to COVID-19 onset, did not live with their parents, and spoke English or French were recruited through the first author’s social media platforms and with assistance from community organizations that serve youth. Numerous participants indicated during the interviews that they had moved to the city in recent years, either from other rural or urban centres within Manitoba, or from other countries, to pursue further education or find employment, as is often part of this life stage transition. Interested participants were contacted by study personnel to explain the study and arrange for the interview.

Demographic forms were completed by participants followed by semi-structured interviews conducted by trained research personnel under the supervision by the first author. Although a sample size of 40 youth was proposed, theoretical saturation was reached at 33 participants [72]. Interviews took place in-person or virtually (audio/video conferencing, phone) depending on participant preference, each lasting 40 to 75 minutes.

Participants who took part in the first interview were invited via email to participate in a graphic recording focus group to further validate the interview findings (member checking) and provide visual representations that contribute to a greater understanding of the findings. Four female YW of mixed ethnicities participated virtually in a 1-hour graphic recording focus group in June 2022 led and conducted by the first author. A graphic recording artist documented the conversation.

### Data analysis

Data analysis occurred simultaneously with data collection. Specifically, the inductive approach of thematic analysis was applied by identifying recurrent themes across participants [73–75] which were organized into a table of contents in Microsoft Word. First the interview transcripts were read and re-read to get a sense of the data and overall meaning. This was then followed by delineating units of meaning from the data, clustering units of meaning to form thematic statements and extracting themes [73–75]. All data were reviewed repeatedly for significant statements by authors RLW, CAI, PT, and AB in an attempt to understand participants’ lived experiences and meanings through themes [73]. Any discrepancies or uncertainty of themes were resolved via discussion among all three authors until consensus was achieved.

Preliminary analyses from the interviews were discussed with focus group participants which helped to uncover new and/or support identified themes. The graphic recording served as a visual representation of the text-based findings and informed the themes emerging from the data. By relating the visual data to the corresponding transcripts, we gained a greater understanding of YW experiences. Demographic data was analyzed using SPSS*® Version 27*.*0* [76].

In a second level of analysis, themes were applied to Frank et al.’s [77] conceptual framework on the social determinants influencing work, health, and well-being. This conceptual framework places employment conditions within their social context (*socioeconomic and political conditions*), which then affect the *social position* of the worker, their *employment conditions*, and their *working conditions* [77]. Interacting with working conditions is *individual susceptibility*. These factors affect the *social and health regulatory and benefits system*, and, then all lead to the resulting inequities in health and well-being [77].

In addition to validating themes via the focus group, measures applied to enhance the study’s methodological rigour included prolonged engagement with the data, careful line-by-line analysis of the interview transcripts, and detailed memo writing [73–75].

### Ethical considerations

This study was approved by the Education/Nursing Research Ethics Board (ENREB) of the University of Manitoba, Canada (Protocol # E2020:073 [HS24324]). Written and verbal informed consent was obtained from all participants, who were also made aware that any publications resulting from the study would not include identifying information. Confidentiality and anonymity were ensured by assigning to each participant a code which was used to identify the interview and focus group transcripts. Participant confidentiality was maintained during the graphic recording focus group by reminding participants to share only what they were comfortable with and asking that participants keep in confidence that what is shared during this session. All study personnel, including the graphic recording artist signed an oath of confidentiality. In this manuscript, participants are referred to by assigned code numbers or referred to as “young workers (YW)”.

## Results

### Participants

A total of 33 young workers, ages 18–26 years, participated in individual semi-structured interviews [33] and a graphic recording focus group (4 participants from individual interviews). Participant characteristics are detailed in Table 1. Additional analysis determined that 28 (84.8%) of the 33 participants identified as BIPOC, of whom 16 (57.14%) had college diplomas or university degrees. Of these 28 participants, 23 (81.14%) were working in sales and service/food or trades sector jobs, and 19 (67.86%) were attending school, college, or university. In contrast, most (4; 80%) of the White/Caucasian participants held a college diploma or university degree, and 40% worked in sales and service/food or trades sector jobs. Exploring by gender-identity, of the 22 (66.66%) females, 19 (86.36%) were BIPOC, 13 (59.09%) had a college diploma or university degree, and 15 (68.18%) worked in sales and service/food or trades jobs. In contrast, of the 11 (33.33%) of male and non-binary-identifying participants, most (9; 81.82%) were from BIPOC groups, 63.64% (7) had a college diploma or university degree, and almost all (10; 90.91%) worked in sales and service/food or trades sectors.

**Table 1:** Participant characteristics.

Young Workers (n = 33)		
**Age (mean = 24)**	Frequency	Percent
18–23	11	33.33
24–26	22	66.67
**Sex or Gender Identity**		
Female	22	66.67
Male	10	30.30
Non-binary	1	3.03
**Ethnicity**		
*Black (includes Black, African American, African Canadian)	15	45.45
*Asian (incl. Southeast Asian, Asian, East Indian, Filipino), & Latinx	10	30.30
*Indigenous (incl. First Nations, Metis, Inuit)	3	9.09
White	5	15.15
**Attending school, college or university**		
Yes	11	33.33
No	22	66.67
**Education**		
High school diploma, Some high school or less	6	18.18
Some college or some university	8	24.24
College diploma	7	21.21
Bachelor’s degree	12	36.36
**Occupation Category** (NOC Level)**		
Natural and applied sciences and related (2); or Health (3)	4	12.12
Education, law and social, community & government services (4); or Art, culture, recreation and sport (5)	4	12.12
Sales and Service (6); or Trades (7)	16	48.48
Sales and Service (food, accommodation and tourism) (6)	9	27.27
**Unionized work**		
Yes	10	30.30
No	17	51.52
Missing or Unsure	6	18.18
**Health Benefits**		
Yes	13	39.39
No	17	51.52
Missing or Unsure	3	9.09

*BIPOC (Black, Indigenous, People of Colour)

**In Canada, occupational structures are based on the National Occupational Classification (NOC version 2.1), a hierarchical structure of job classifications that includes 10 general occupational categories ranging from the highest, level 0 (legislative and senior management) to lowest, level 9 (manufacturing and utilities), many of which require formal education [71].

### Themes

#### 1. Pre-COVID realities exacerbated by COVID-era challenges

Although interview and focus group questions were focused on COVID-Era and future timeframes, YW also spoke considerably about their pre-COVID-19 employment experiences. Clearly evident from these discussions was that COVID-Era challenges have amplified pre-COVID-19 realities across multiple areas of YW’ lives. To demonstrate these impacts, Frank et al.’s [77] framework of social determinants influencing work, health, and well-being is used to present the themes, where ‘Pre-COVID Realities’ provide a baseline for ‘COVID-Era Challenges’ themes (Table 2).

**Table 2:** Social determinants of health pre-covid & covid-era themes.

Social Determinants (Frank et al., 2023) [77]	Pre-COVID Realities	COVID-Era Challenges
Socioeconomic and Political Conditions	• Economic Uncertainty	• Economic Security/ Insecurity
Social Position	• Racism & Discrimination • Differing Social values ○ Society ◾ Generational Divide • Feeling unvalued as young people	• Racism, Inequity & Discrimination • Differing Social Values ○ Society ◾ Generational Divide ◾ Feeling undervalued as young people & front-line workers ○ Workplaces ◾ Feeling unheard, undervalued, and uninformed by employers
Employment Conditions	• Precarious Employment ○ Limited Full-Time jobs ○ Employment Insecurity ○ Influence of Automation & Technology ○ Normalization of Precarious Employment • Income and Income Distribution ○ Low Wages ○ Higher wages with Permanent Employment	• Precarious Employment ○ Increased Hours & Workload ○ Reduced Hours & Work Opportunities ○ Future Employment Uncertainties • Income and Income Distribution ○ Frontline Workers & Minimum Wage Earners
Working Conditions	• Limited Work Breaks & Paid Vacation • Supportive Workplaces	• Safety: Working From Home & Frontline Work • Supportive Workplaces
Individual Susceptibility	• Coping mechanisms • Life Course Transitions ○ Independent Living ○ Entry into Workplace ○ Financial Autonomy ○ Emotional Self-sufficiency ○ High School-to-University/Work Transitions	• Positive Coping Mechanisms, Resilience, and Hope • Young Workers’ Personal Values Valuing self and health more than systems and workplaces • Life Course Transitions ○ Navigating the Pandemic while Adjusting to Independence, Autonomy & Self-Sufficiency ○ Without Family Resources/Supports
Social and Health Regulatory and Benefits Systems	• Lack of Health Benefits & Paid Sick Leave	• Lack of Health Benefits & Paid Sick Leave • Unemployment Benefits/CERB

#### 2. Compounding impacts of COVID-era challenges on the mental health of young workers

Participant quotes from interviews and the focus group are presented below under the six main COVID-Era themes (social determinants influencing work, health, and well-being) [77]: socioeconomic and political conditions, social position, employment conditions, working conditions, individual susceptibility, and social and health regulatory and benefits systems. Notably, these COVID-Era employment-related challenges were compounded through the different layers of social determinants, impacting YW’ MH.

**Fig 1:**
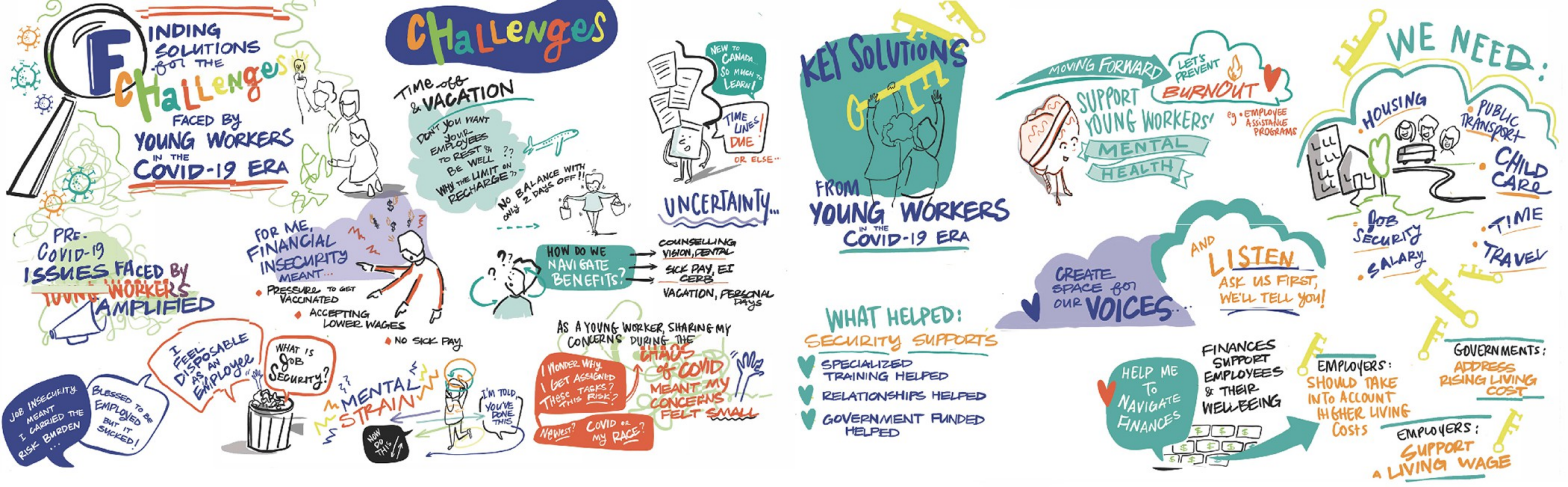
Graphic recording focus group approximately here.

The graphic recording image (see Fig 1) from the focus group further supports and echoes the Pre-COVID and COVID-Era interview findings. Text phrases in the image are paraphrased quotes from focus group participants. Within the image reading from left to right portrays YW’ pre-COVID realities, then COVID-era challenges, and ending on the right with YW key solutions for action directed towards government and employers.

### Socioeconomic and political conditions

Approximately one in four YW spoke of economic security during COVID-19, due, for example to, saving money due to financial assistance from family, or increased opportunities for more shifts/work hours. Still, a YW noted that even though, “*financially I was stable* … *emotionally it did a take toll on us*.*”* [YW_28 Asian/Latinx Female].

Many more participants, however, shared about the significant challenges they encountered with economic insecurity (e.g., wage reductions) due to pandemic-exacerbated employment precarity e.g., reduced work hours or job loss. These challenges left some YW struggling, having to resort to a line of credit and credit cards to pay bills, resulting in stress and emotional strain.

***YW_27:*** *“COVID 19 has really affected most of the people, not only me, but most of the people mentally… because of the financial instability which COVID 19 really caused on it. … And it has really been consuming my mind and I’ll say it has not given peace and that really affected me mentally because it’s giving me a lot of stress, and pressure.”* [Black Male]

### Social position

Stories of racism and discrimination in the workplace continued during the COVID-Era, exacerbating pre-COVID realities. These behaviours were both observed, “*just like a lot of racism*, *a lot of homophobia*, *a lot of Sinophobia…”* [YW_08 Black Female] and experienced by YW:

***YW_22: “****I felt very threatened … myself and another colleague who worked different positions in the same team were both black minorities and were the only two people being forced to work in the office for four months… What’s shocking was that employees that were hired after me to help with our backlog were given laptops to work from home right off the hop. And it took me a year and a half to get a laptop.”* [Black Female]

Adding to pre-COVID realities, the differing social values across generations and expectations of productivity was more evident during the pandemic, further contributing to *…*

***YW_22:*** *“…a divide, because the older generation is expecting a certain amount of work to be pushed, whereas the younger generation that is closer to my age who are in higher roles understand, like they have more compassion, they understand burnout more…****”*** [Black Female]

These generational differences carried over into YW and minimum wage earners feeling undervalued by society and government in the COVID-Era.

**YW_18: *“****I’ve been a minimum wage worker all of my working life and I’ve sort of learned that I’m just really not respected or as important … we do all this essential stuff, like people want to go to the drive through and grab a coffee. That’s a minimum wage worker. … I honestly think like minimum wage workers are the backbone of our society and they are just completely forgotten about*. …*I don’t think the government did enough … I don’t think they understand enough of how important we are and how little we get rewarded… I like work to my maximum capacity… and I’m given pretty much minimum back, like I’m, I’m given nothing… and it feels very unfair*.” [Asian/Latinx Female]

Participants called to be acknowledged for their efforts and contribution to society, as poignantly expressed below:

**YW_15**: “…*I don’t think they* [government] *put much thought into like students… Or like young workers. …, we’re still part of society, we still work, we still contribute, … I think we’re just really; we’re just seen as kind of like ‘oh like they’re not really contributing’ opposed to like seniors and like older workers. I think like we get kind of forgotten. … like but what about us… Like the ones who work retail that weren’t able to work from home, who are in school. Like it’s I don’t know we’re just so, I feel like we’re just, some of us kind of fall through the cracks*.” [Asian/Latinx Female]

Yet during the pandemic it was YW who *"… are just going to work*, *which is also what young people have been asked to do is to restart*, *like help in restarting the economy…”* [YW_05 White Female]. Further intensifying YW’ feelings of being undervalued by other generations was the lack of recognition of the emotional impact the pandemic had on YW:

***YW_22: “****I think they* [older generation] *see it and they don’t value like the importance of what we’re going through. … I just feel like the older generation just feels like it’s, like we’re, we’re living the life, like we’re just enjoying ourselves, it’s fine, we’re, we’re working from home so we’re not complaining. But no, we’re actually not okay, we’re all depressed. Nobody care[s].”* [Black female].

Similarly, at their workplaces YW’ sentiments of feeling unheard, undervalued, and uninformed by employers during the pandemic were strongly articulated. The excerpt below builds on the COVID-Era challenges identified above, and makes further comments regarding YW’ contribution to businesses and communities during the pandemic, yet still being underappreciated:

**YW_12:** “*…we are the ones who stepped up and we, we did the things that was supposed to be done… So, we should have given like more than wage*[s]*, … I would have liked like some health benefits as well* … *I think like they should appreciate us … Because we’re working to support the store and to support the community as well…”* [Asian/Latinx Male]

### Employment conditions

In addition to economic challenges, the onset on the COVID-19 pandemic set a myriad of employment changes in motion for many YW, including precarious employment, e.g., increased workload, reduced hours, lay offs, or job losses.

While some YW appreciated the economic security that came from opportunities to work additional hours, approximately half of the participants spoke of having increased workloads and greater demands from employers when the pandemic began. YW spoke of having little to no choice in taking on extra shifts or overtime due to being “*constantly understaffed*” and feeling obligated to work extra hours but not being adequately compensated.

***YW_19: “****… so you’re supposed to go for that shift because many people are laid off during COVID, …it changed a lot because you, you get that you are working more hours, you are getting tired, but you don’t have any days off or you don’t have enough time to rest… And then also the business is not that good because even the pay that you are getting is just menial and just little and you’re working so many hours”* [Black female].

Not surprisingly, working additional hours or *preassigned amount of overtime* adversely impacted YW’ MH, resulting in burnout or having to take “*short-term disability because it’s just too much”* and “*…I also can’t sleep because I’m so stressed…*.” [YW_22 Black Female]

Many participants who were previously working full-time had reductions in their hours or salary, typically with no choice or alternatives provided by their employers, such as a participant who shared that, *“we are either to accept either a* [salary] *cut-off or you could resign*.*”* Several other YW shared experiences of being suddenly “*like totally jobless”* or “*I was literally working on the last day when our manager and was like yeah so we’re not opening tomorrow*, *so like you guys are being laid off”*. For YW who were on student and/or work visas, they faced other unique challenges with continued employment, given national slowdowns on processing work permits during the pandemic, leaving one YW discouraged; ***“***… *So*, *all these years of coming here*, *studying*, *looking for work*, *it’s all*, *it’s all in that*, *like it’s*, *I don’t have the words*, *hmm*, *it’s all jeopardized*, *…*.*”* [YW_03 Asian/Latinx Female]

One YW succinctly captured the compounding impacts of these experiences, “*when you get laid off*, *that’s a crisis and that could be*, *well first of all that’s a financial crisis and that could be also a mental crisis*.” [YW_33 Asian/Latinx Female]

YW additionally associated pre-COVID employment precarity with COVID-era employment insecurity and future employment uncertainties. For example, “…*there’s always been a lack of job security I found just in my life*. *And that like the pandemic really just increased that*” [Focus Group]. With future employment opportunities deemed even more uncertain *…* ***“****it’s going to be harder to find a job than it already was*.*”* [YW_06 White Female]

Further participants raised issues regarding income and income distribution. They noted that it was primarily lower wage earners and ‘essential workers’ in front-line retail and hospitality jobs who had to continue working face-to-face with customers and clients during the height of the pandemic, raising issues of inequity, and not feeling acknowledged for the sacrifices and risk they were taking on.

**YW_18: “**I don’t think the government did enough [for minimum wage workers] …. I don’t think they understand enough of how important we are and how little we get rewarded… I’ve always said like I’m a minimum wage worker and I, I like work to my maximum capacity… and I’m given pretty much minimum back, like I’m, I’m given nothing… Um and it feels very unfair.” [Asian/Latinx Female]

### Working conditions

Regarding pandemic employment and working conditions, some YW who worked from home appreciated the flexibility and felt a sense of relief and safety as they did not work in direct contact with people. Conversely, those who were mandated to work from home shared challenges with staying on task, and work-life balance “*it’s pretty draining to work exclusively from home and to like have that boundary between home and work life*, *like so blended*.” [YW_05 White Female] Participants further shared that not being able to meet with friends and family in-person, **“…***depressing being at home all the time…”* [YW_03 Asian/Latinx Female] For some young people the compounding challenges of working from home, depression, and anxiety, led to *using drugs* or alcohol:

***YW_05:* “***I started working from home in March…*. *The first few months were great… But I would say within the last four months I’ve been like having major anxiety, low productivity, depression. The switch was wow I have too much freedom, this is not good… I can roll out of bed, and it just didn’t feel good rolling out of bed and just working in pajamas… And then I lost my schedule… And then I started drinking*.” [White Female]

Many YW however were in frontline jobs that continued during the height of the pandemic and were put in situations where they were in direct contact with customers/clients leaving them feeling unsafe, for example:

***YW_28:*** *“At the start it was very difficult because some customers who didn’t wear masks at all, and they were not following any regulations, … it was very risky to work during that time, but as we have to work, we can’t skip jobs, ….”* [Asian/Latinx Female]

In contrast, while less common, some YW did feel supported and heard by their managers who prioritized employee well-being and encouraged taking sick time if needed and vacation.

***YW_01: “****I think what makes it positive is my employer really puts the well-being and care and consideration of their employees first and I know that is a rare situation. So, we are encouraged to take vacation, you’re encouraged to still take like sick days if we’re unwell, we’re encouraged to kind of put ourselves first and I feel like when an organization focused first on the well-being of their employees, they perform a lot better.”* [White female]

YW in supportive work environments also spoke of opportunities for advancement and stability in their jobs or did not feel productivity pressures, which along with having supportive coworkers, contributed to a positive work experience.

### Individual susceptibility

Despite the myriad of challenges experienced during the pandemic, some YW demonstrated strength and positive coping mechanisms to mitigate the numerous stressors and MH challenges they were experiencing. For example, connecting with family and friends in-person or via social media, listening to music, meditation, interacting with pets, being outdoors, or “… *travelling or maybe I just go for a walk and maybe cycling*, *that was really something that was helping me*… [YW_26 Black Female] Several YW also spoke about how they had reflected on their experiences, sparking new motivations, *there’s so much more that I’m going to do as opposed to being like I’ll do it next time (chuckle)*. [YW_17 Indigenous Female] and *“through COVID it has made me to even be stronger… that motivation of even working harder and knowing that you have to be ready for anything*, *… “*. [YW_19 Black Female] Finally, resilience and hope was evident amongst this cohort who are key contributors to future economies.

**YW Focus Group**: *In spite of everything*, *I still believe that there’s still so much good in the world*. *We’ve made progress just ever so slightly*. *But there’s a quote from my favourite anime*, *Cardcaptor Sakura*, *that I have always stubbornly held onto*. *“****絶対大丈夫だよ***,*” which roughly translates to*, *“everything will definitely be alright*.*”*

Despite these challenges, the personal values of YW allowed them to take steps towards self-care, healthy boundaries, and valuing themselves to avoid appeasing productivity or employer expectations, preferring “… *more like a work to live sort of thing… rather than like a live to work…”* [YW_18 Asian/Latinx Female], and,

**YW_08**: [Participant did not return to workplace post-COVID] **“***I value myself more than I value contributing to a system that’s already broken. Like I value my, I would rather be healthy than and end up being sick and end up in the hospital right back where I shouldn’t have been you know.”* [Black Female]

These personal values of YW also guided their approach to work-life balance and choices, within the constraints of current structural environments and economy.

**YW_22**: “*It’s more about what you love now more than it was, just making money and providing for a family, because so many of us can’t afford to have families right now in this age because of what’s going on in the world*.” [Black Female]

Life course transitions such as moving to a new city for work or university/college after high school graduation, entering the workforce, living on their own, or being independently responsible for earnings, school, and jobs were a complex reality for numerous YW. For newcomers, these times could be particularly taxing:

***YW_12:*** *“I have never lived by myself in my life because like I used to live with my family, so like I didn’t know like the coping, … Like how to support yourself… So, it was the biggest challenge for me… like emotionally, financially, so I had to do like, like everything else, …* [in home country] *I was only focused on my studies.”* [Asian/Latinx Male]

Other YW shared frustrations with school-to-work transitions, such as obtaining employment within the field they were trained for after completing university, leaving some YW having to choose to “*do any job”* or to incur the costs of pursuing further education.

The COVID-19 pandemic exacerbated the challenges that came with this life stage, especially for participating YW as they were not living with their parents or families and thus often without family resources and nearby support at the time of the interviews.

**YW_22: “***And I was also living on my own and going through it all* [COVID-Era]… *And so I was taking like the bare minimum three courses each semester just so I could get by and not overwhelm myself while, while also working three jobs. … like I’m finished [undergraduate degree] and if I want to continue, I really need to be able to pay for it… Because the last thing I want to do is put myself in debt… Especially when there’s sort of no one else I can rely on…”* [Black Female]

### Social and health regulatory and benefits system

Given the workplace challenges many YW were experiencing during this era, health benefits and paid sick leave became critical. However, less than half (n = 13; 39.39%) of participants had workplace health benefits and paid sick leave (Table 1) yet felt they had no choice but to work even if sick due to the economic impact, “*… I don’t get [sick pay]*, *I just lose the hours… you would just work until maybe you are so sick… Yeah*, *you can just do those hours*.*”* [YW_24 Black Male] Not only did lack of paid sick time impact YW’ economic security, but it also impacted their MH. It was “*a stressful situation… I was like worried that I wasn’t going to have any sort of income if I was sick… Because if I was sick for a week then that’s a whole week without pay*.” [YW_17 Indigenous Female]

With the onset of the pandemic in Manitoba many of these young workers in minimum wage positions were laid off by their employers, with little to no assistance as to how to access government unemployment benefits, as shared by a YW who was newer to Canada.

**YW_33**: “*Yeah, so the pandemic hit on March 2020 I guess yeah 2020. And that was March 2020 and then the last day I went to work it was March 14 … and now we were laid off. I didn’t know how EI and all of those programs worked. So, I had to search by myself, ‘cause no one, no one explained it to us, no one told us how to do it. I had to do all the searching*.” [Latinx Female]

The Canada Emergency Response Benefit (CERB) in particular was a topic discussed by many YW. They shared that in most cases, these (and other government) benefits were either difficult to secure due to limited availability of online information and support, website navigation issues and application instructions, or eligibility criteria restrictions, such as having a second job. YW also noted that the financial support received from various pandemic-related government benefits was often considerably less than what their income was prior to the pandemic or benefits were given as a one-time payment leading to financial and employment uncertainties.

**YW_29:** “*My workplace told me, like they sat me down and said, okay you’re going to be on CERB but you’re going to come back in I think it was three or six months … ‘cause we, no one expected COVID to, to go on this long. So, I was expecting to be brought back. And then they called again during I think it was in the summer, somewhere between July and August where they said they were going to extend me on CERB, so then it was another few months until they called me again, I think September/October and they told me that they were uh they had to let me go*.” [Asian/Latinx Female]

**Fig 2:**
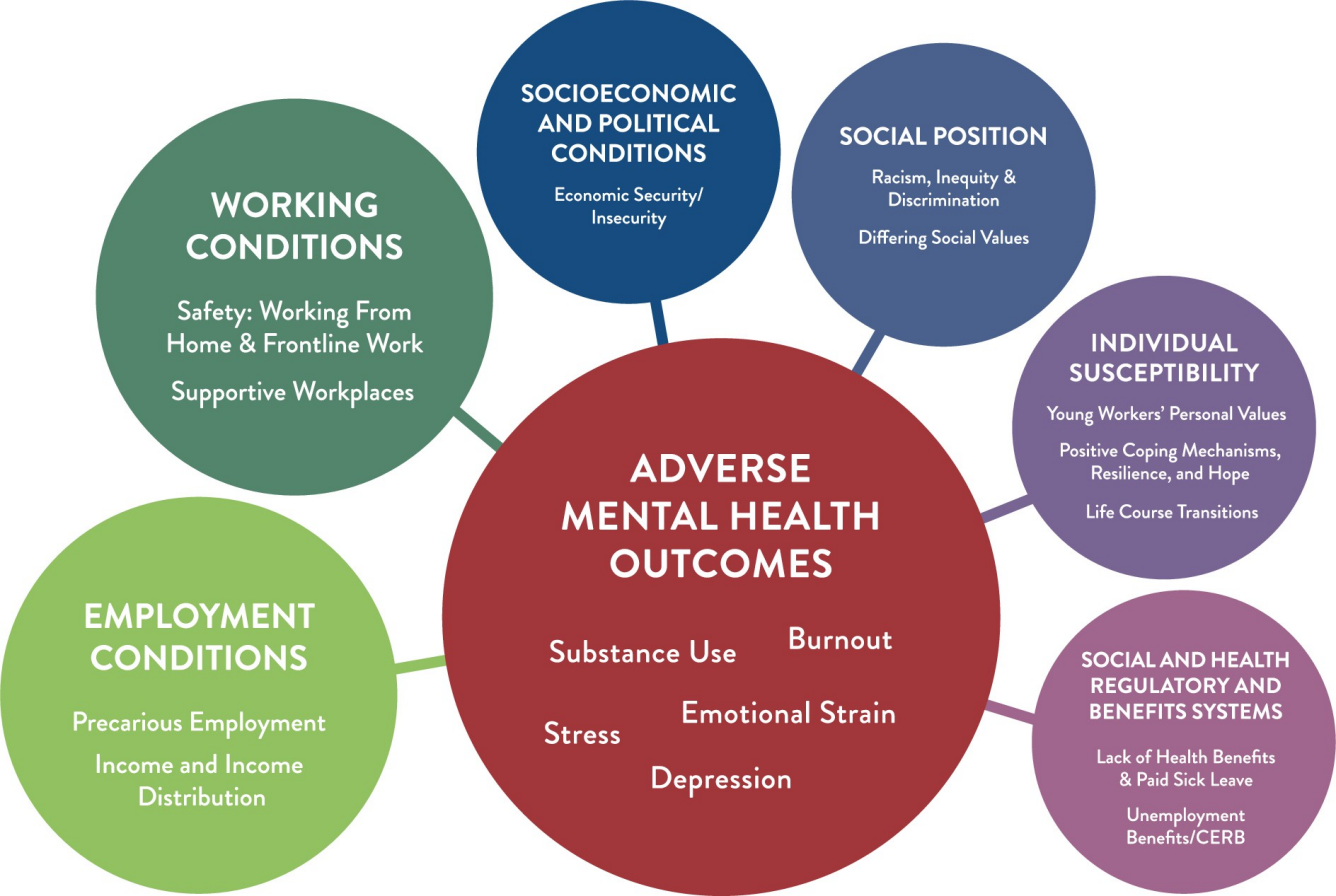
Compounding impact of the social determinants of health and covid-19 on young workers’ mental health approximately here.

To summarize, the compounding impacts of the pandemic on the MH of YW is shown in the diagram in Fig 2. The size of the circles (themes) in Fig 2 reflects how much these different social determinants were discussed during by participants, e.g., larger circles equal greater concerns among YW. This diagram visually shows that Employment Conditions (light green circle) and Working Conditions (dark green circle) had the greatest impact on YW’ MH (red circle) during the pandemic, with Socioeconomic and Political Conditions (dark blue circle), Social and Health Regulatory and Benefits Systems (pink circle), Individual Susceptibility (purple circle), and Social Position (slate blue circle) having slightly less, but still significant influence.

### 1. Moving forward: Young workers’ recommendations for action

Following our ‘youth as experts’ approach, recommendations gleaned from this study are those of the YW participants. Table 3 (interview participants) and Fig 1 (focus group graphic recording) present recommendations that address YW’ Pre-COVID realities and COVID-Era challenges. Recommendations in Table 3 are organized according to Frank et al.’s [77] conceptual framework. While participants presented recommendations that address each component of this conceptual framework, most recommendations focused on improving aspects related to YW’ social position and strengthening social and health regulatory and benefits systems.

**Table 3:** Young workers’ recommendations for action.

Themes/Sub-Themes	Recommendations		Example Quotes
	For Employers	For Policy-Makers/ Government	
Socioeconomic and Political Conditions	• Support a living wage	• Address rising cost of living • Provide financial services and guidance to assist navigating financial challenges and making informed financial decisions	***YW_21*:** *“Yeah*, *I would love them [employers and policy makers] to know that especially the young people*, *it’s really getting difficult for us to get good jobs… you feel like you*, *you might be having everything that that job entails*, *but still*, *you fail to get the job because you don’t have the connection…”* [Black Female] ***YW_FG1***: *…just some guidance financially ‘cause you don’t know how to handle things when you don’t have like an adequate like income… if I don’t have a raise then what’s going to happen with my daily*, *you know my daily activities*, *whether it’s rent*, *food*, *like gas*, *and all that*.” [Females, mixed ethnicities]
Employment Conditions: Precarious Employment	• Provide permanent, stable work opportunities for young people	• Provide permanent, stable work opportunities for young people • Improve efficiency of work permit approvals and renewals for international students/workers	***YW_19*:** *“I think if at least if people are given opportunities to even work in more stable jobs or permanent jobs*, *that would really change a lot for the young people… And they will be motivated to even give the country a better look*.*”* [Black Female]
Social and Health Regulatory and Benefits Systems	• Provide Health Benefits (incl. Paid sick leave) & safe work equipment (e.g., PPE) • Develop, implement & maintain: • Health and safety representatives in the workplace • Accessible mental health policies in the workplace	• Provide micro-level funding (for e.g., paid sick leave, childcare, student and mental health supports) to improve macro level outcomes • Develop and share mental health education and coping strategies for young people	***YW_24*: *“****…I don’t think the government is uh is doing the best to*, *to help young people to deal*, *to deal with mental health*. *I think my opinion it* [pandemic] *has affected many youth*, *many young people… So*, *the government should*, *should try to*, *to find a way in which to*, *it will*, *it will educate the young people about mental health… And how to cope with the stress and such*.*”* [Black Male] ***YW_08*:** *“Actually*, *follow the policy and protocol when somebody says they need a mental health day or that they need to uh*, *yeah like actually ‘cause there is policy and protocol involved um with that*, *but it was not followed by like the manager and there’s stigma still so people are reluctant*. *So just follow it and maybe you need some training because you still have stigma against mental health*.*”* [Black Female]
Social Position	• Use fair and equitable hiring practices, including equity, diversity & inclusion (EDI) practices (e.g., avoid nepotism & ageism) • Develop, implement & maintain: • Accessible racism, EDI, and discrimination policies		***YW_04*: *“****I wish there was some sort of like*, *they’d send out some sort of protocol like what to do when your co-worker receives a remark*, *like some sort of bystander training*, *like*, *or just like a wide statement or like here are these resources or if your co-worker receives some sort of um you know remarks from clients*, *like this is what we can do and here’s the procedure*, *there was nothing really like that*.*”* [Asian/Latinx Female] ***YW_08*:** *“Well here’s something that tends to work*. *Um have a policy that actually gets implemented for hiring uh ethnically*, *ethnic*, *religious and like*, *like um and also diverse like bodied people into different units and to make sure that’s actually happening*, *… And there’s nothing wrong with holding these positions but there needs to be more ethnically diverse… like it can’t just be white men and women*, *it’s just not going to work*.*”* [Black Female]
Social Position	• Demonstrate value, understanding, respect & support for young workers • Create space for voices of young people	• Acknowledge and prioritize young people and their unique contribution to society	***YW_18***: *Like what*, *what needs to be done*. *I feel like honestly it should be common sense that you know like these people who are working around the clock*, *working paycheque to paycheque to provide for themselves like I don’t know they’re doing so much and they’re helping so much with the overall like economy and um I just don’t understand why people can’t understand how important they are*. [Asian/Latinx Female] ***YW_19*:** *“I wish they [companies] just knew how important a worker is to an organization… Because the way*, *the way you treat a worker determines what profits you make*, *if you treat them good*, *they’ll even be happy to even operate the business in a better way than you would even expect… I think it would be helpful if organizations [companies] are able to treat those young people in a better way…”* [Black Female] ***YW_27*: *“****I’ll say the institutions*, *the organizations*, *the companies uh I’ll say they should treat their workers equally and uh really like respect their grievances if the workers have really like placed an issue*. *They should be listened to and the*, *the issue really like uh be considered*, *…”* [Black Male] **YW FG1**: *“Just like even being heard as an employee makes us feel more valued … if we could be heard as employees that would be a lot better than decisions being made for us that affect our lives directly*.*”* [Females, mixed ethnicities]
Working Conditions & Individual Susceptibility	• Develop ‘basics of adulthood’ workshops, programs, and skill development opportunities accessible to young people of any age • Provide mentorship to newly employed young workers	• Create life-course focused career training & skills development programs and relevant employment creation schemes	***YW_20*:** *“I just feel like um we are going through much and even lots of people might not be aware of that so I feel like the government especially for this time since things are quite difficult if they could be able to provide like a training uh program… The young people can be equipped with skills in different fields so that they can be able to like uh not depend uh so much on being employed yeah… So*, *if they are able to like sort of provide funds so that*, *so that the young people can be equipped with their skills so that they can be able to like uh start their businesses and*, *and be able to like stand alone and just depend*, *depend uh for themselves…*.*”* [Black Female]

## Discussion

Our study highlights the compounding impacts of structural, employment, and social/community determinants that influenced YW’ lives pre-COVID, which were exacerbated in the COVID-Era and continued to impact their MH and well-being. Furthermore, the arts-based graphical recording of the findings is novel within health and labour-focused research. Since focus group participants could see the key points being raised as the artist was drawing, this helped to engage them in the discussion and participate in the analytic process. Use of arts-based methodology in collaboration with YW aided in reinforcing findings from the individual interviews, and validating final key themes. By using a social determinants of work, health, and well-being framework, this study provides a glimpse of how the compounding impacts of the pandemic affected the ‘whole person’ of participating YW. While qualitative studies exploring the impact of COVID-19 on the work and lives of YW in Australian [3] and U.K. [79] exist, to the best of our knowledge, this is the first such qualitative, arts-based study in Canada.

### Socioeconomic and political conditions

Young workers situated their employment experiences during pre- and COVID-era timeframes within broader social determinants, such as socio-economic structures, the labour market and the economy, similar to limited economic opportunities identified by youth during development of Canada’s Youth Policy [14]. This demonstrates that Canadian young people are keenly aware of these structural contexts and are articulating how these structures are adversely impacting their employment and health. As noted in Canada’s Youth Policy, the Government of Canada does offer various initiatives to support skills development for youth to obtain the required skills of the rapidly changing labour market [80]. However, with even greater labour market challenges and instability since the pandemic, it is critical that these programs meet the needs of young people. These challenges could be addressed through implementation of recommendations by YW to support a living wage and address the rising cost of living, and through improved alignment of training with labour market needs as per employment objectives of Canada’s Youth Policy [14].

### Employment and working conditions

Employment and working conditions, two factors that affect inequities in health and well-being [77, 81], were some of most impactful determinants on YW MH during the COVID-era, as evident in Fig 2. For many participants in our study, low wages, along with precarious employment [14], both before and during the pandemic, was connected with economic insecurity in the present and economic uncertainty for the future, similar to UK minority youth [82]. Our findings that YW employment experiences and working conditions negatively impacted their MH supports Frank’s [77] social determinants framework, and similar. For example, the Dahlgren and Whitehead model states, “differences in the physical and psycho-social working environment constitute one of the main determinants of socio-economic inequities in health” (p. 20) [81]. Working conditions that are present are often tied to both salary and education levels, with more unhealthy working environments existing for workers who have a lower salary and lower education level [81].

Interestingly, this generation has been advised that post-secondary education is the pathway to better jobs [17]. However, this did not seem to be the outcome for most (27/33; 81.82%) participants who had or were completing post-secondary education and were employed outside of their area of education, which is in contrast to Dahlgren and Whitehead’s social determinants framework [75]. To explain, firstly, the high number of participants working in sales and services, including food and trades occupations (25/33; 75.76%) which are typically lower paying could be expected to a certain extent as YW are new to the labour market and early in their careers, or may be continuing their education and working part-time. Nonetheless, while these types of jobs provide employment and work experience opportunities [83] they also typically lack stability and health benefits, which leaves YW in vulnerable positions. Secondly, these findings may be influenced by other social determinants of health factors such as immigration, race, or Indigenous ancestry [84], further exacerbating inequities. Similarly, in 2017, the Canadian Expert Panel on Youth Employment identified this concern, noting that some young Canadians such as Indigenous and recent immigrant youth struggle more with finding employment due to structural determinants of health including colonization and discrimination, or to limited community and family connections and resources [12], similar to some participants in this study. In addition, Raphael et al. further note that “new Canadians are frequently unable to practice their professions due to a myriad of regulations and procedures that bar their participation” (p.42) [84]. Regardless of the reason, as part of Canada’s Youth Policy objectives regarding employment, the Canadian Government committed to assisting youth to overcome employment barriers and to applying principles of equality, such as Gender-based Analysis Plus (GBA+) to help them reach economic success and further strengthen Canada’s economy [80] which is required to provide more equitable employment for YW.

There were clear issues emerging from the findings related to job quality and a sense that employment and working conditions are worse for young people since the onset of COVID-19, exacerbated by the absence of paid sick time and health benefits. Previous research has established the relationship between poor working conditions and health outcomes, with implications for long-term conditions, e.g., hypertension and cardiovascular disease, but these conditions are also connected with current social and economic determinants [85]. Furthermore, job strain, evident in YW’ stories, has been previously identified as a key health-related dimension of the work environment, which “exists when people’s autonomy over their work and their ability to use their skills are low, while the psychological demands placed upon them are high” (p.175) [85]

The Psychology of Working Theory (PWT) [86] aligns with our findings and may assist in explaining some of our findings as the PWT purports that work is considered inextricably linked to and inseparable from the worker, including racial identity and cultural background, and “is largely focused on disparities in access to decent work across social contexts and identities” (p. 238) [87]. According to the ILO definition of decent work there are minimum standards required to adequately function at work; [88] including 1) physical and psychological safety, 2) adequate access to health care, 3) adequate compensation, 4) adequate free time and rest, and 5) organizational values that support one’s [own and] family values [86]. It is evident from our study findings that many YW participants were not afforded decent work either before or during COVID-19. This is critical as “performing decent work leads to *need satisfaction*, *work fulfillment*, and *well-being*”,(p.128) [86] which all impact MH. Consequently, if young people are not able to participate in decent work it intensifies the difficulties faced by YW pre- pandemic.

### Social position

As a determinant of health “social exclusion creates a sense of powerlessness, hopelessness and depression that further diminishes the possibilities of inclusion in society” (p. 42) [84]. Those who are likely to experience social exclusion include Canadians who are BIPOC and newcomers [81, 84]. People in these social positions are more likely to be lower wage earners, unemployed and have poorer access to educational, social and health services [84]. Social position may have played a role in access to education and employment for BIPOC participants in the current study, where slightly more than half (57.00%) of the 28 BIPOC participants had college diplomas or university degrees, and most (82.14%) of these were working in lower wage earning sectors such as sales and service/food or trades. Based on our study findings we contend that YW are amongst those more likely to experience social exclusion, and that the intersecting factors of social position (e.g. ethnicity, gender, age) impacted YW’ employment experiences and compounded adverse mental health as explained in the following paragraphs and depicted in Fig 2.

According to the World Health Organization (WHO), COVID-19 has accentuated racism, and xenophobia [89]. Similarly, for Canadian YW in our study, the pandemic exposed and worsened these experiences in the workplace. Similar to minimum wage earning young workers participating in other studies and YW in this study who kept the economy going during the pandemic were often also marginalized, racialized workers [51], with the combination of precarious employment and the pandemic having disparate effects on them [90]. For example, most participants who were BIPOC (22/28; 78.57%) were college or university educated. Yet, most (18/22; 81.82%) of these YW were working in sales and service, and hospitality jobs which are lower NOC category occupations and lower paying [78]. Comparatively, the national rate (48.15%) of 15–24 year-old Canadians who were employed during the pandemic (2021) in these same job categories is almost half of the rate amongst study participants [91]. This suggests that there may be compounding racial or discriminatory determinants at play for study participants, which was also shared during the interviews. These findings are consistent with other qualitative research [82] and supports the 1999 ILO call for decent, equitable work for all [92].

Ageism has also been identified as a social determinant of health and form of discrimination, leading to social and societal divisions [93], and resulting in poorer health outcomes and inequities [77, 93]. This was especially noticeable during the pandemic through “…inadequate protection of… young people’s mental health…” (p. 1333) [93]. Although not explicitly stated by YW in the current study, we propose that aspects of ageism towards YW were evident in their workplaces during COVID-19 with respect to precarious employment, low wages and lack health benefits, and not feeling valued by employers, a form of institutional ageism [94]. These factors have been identified in a recent scoping review [95] and in the 2021 WHO Global Report on Ageism [94] as evidence of ageism towards younger people, which may exclude them from influence and power [94]. This also ties to YW’ sentiments of not feeling heard or valued by society and not being included in decisions made by employers that directly affected them during the pandemic and also to social exclusion, a social determinant of health which negatively affects physical and MH and well-being, and can shorten lifespans [89]. While the intent of this study was not specific to ageism, our findings may contribute to the limited body of literature on the impact of ageism on young people.

In Frank’s [77] framework of the social determinants of health, gender plays a joint influential role along with age, race, and social class on work and health. For example, women are predominately employed in caregiving or administrative positions, whereas men more commonly occupy management positions [96, 97]. Our study findings are in contrast to these norms as 90.00% of male-identifying YW worked in lower paying sales and service/food or trades jobs whereas 68.18% of female-identifying YW worked in that same sector. This is despite the finding that 70.00% of male and 59.09% female YW had college or university degrees which usually leads to working in higher paying job sectors. These findings suggest determinants other than gender that relate to social position may be influencing YW’ opportunities for working in their area of training, which may also negatively affect MH.

The stark generational differences in social values, for example, productivity expectations, YW being unwilling to contribute to a ‘broken system’, and YW’ feelings being undervalued both prior to and during the pandemic, is an important study finding. This suggests an unravelling of the social contract between generations. Recently the social contract was defined as “the partnership between individuals, businesses, civil society and the state to contribute to a system in which there are collective benefits.”[98] This breakdown of the social contract is leaving young people unable to be the best version of themselves [80], and to fulfill their potential [99], yet society depends on this generation of YW to develop and support future economies particularly given the aging population [12]. This has significant implications for future workforce fulfillment, pensions, and health costs [12]. Thus it is critical that young people are given space to add their voice [100], and be afforded the right to be heard and respected, as recommended by YW in this study, to be able to reach their full potential through implantation of leadership and impact objectives in Canada’s Youth Policy [80], which benefits all Canadians.

### Implications for policy and practice

Our study findings have implications for policy and practice, for example, full implementation of, and action on Canada’s Youth Policy [80] objectives regarding employment, leadership and impact, and mental and physical health would address the compounding impact of the social determinants of health and COVID-19 on YW’ mental health and wellbeing. Employers and staff working in youth-focused organizations and programs such as career and MH counsellors can also ensure YW are made aware of existing programming, and tailor supports based on YW’ recommendations in the findings.

### Strengths, limitations and future research

A strength of the study is that we engaged with young people, who are experts in their own experiences, and are best able to provide meaningful recommendations to address their employment and compounding challenges. Also, we collaborated with community organizations within a major city and recruited participants from groups that were ethnically diverse, with most participants identifying as BIPOC—groups who are minoritized on multiple axes of social power—giving them voice. Further, most of the participants lived within this same major city where employment opportunities were similar, allowing for comparison of findings across our sample.

While we aimed to recruit a sex and gender-diverse sample, a limitation of this study was that participants were primarily female and living in a major city in one Canadian province. Thus the findings are not representative of YW who are male, non-binary, or gender diverse, and may not be generalizable across other urban or rural geographic regions in the study province, or other Canadian provinces. Also, the focus group and most of the semi-structured interviews were conducted virtually. This may have influenced what was shared by participants through some noted challenges with respect to having a private meeting space for YW to attend the virtual sessions, poor internet connection, and difficulties with rapport development. However, the virtual interview format added an element of convenience for participants by providing enhanced flexibility to attend sessions, as youth may have irregular schedules due to school and/or work commitments.

It would be beneficial to extend the sample to YW in other parts of Canada to further understand the impact of the pandemic on their work and life experiences, particularly aiming for a more gender-diverse sample. Comparison of YW’ experiences for those who had a high school diploma only versus a college diploma or university degree could further ascertain potential differences due to educational attainment. Furthermore, future research comparing Caucasian and BIPOC YW experiences could assist in better understanding how social position influences employment opportunities and MH outcomes. Exploring the perspectives of other target participants, such as older workers, employers, or mental health professionals, could provide a more holistic understanding of the workplace ecosystem and its impact on mental health during the COVID-19 pandemic [101–103].

## Conclusions

Unique findings from this study highlight the perspectives, experiences and challenges of this cohort of YW who reject the ideals of neoliberalism seen through economic insecurity (socioeconomic and political conditions) and precarious employment (employment conditions), and who yearn for social inclusion (social position) and work-life balance (working conditions). It is important that these compounding social determinants are acknowledged and addressed by government, health policymakers, and employers to provide decent work, ensuring a sustainable, mentally healthy Canadian workforce going forward. Furthermore, most of the challenges and pre-existing inequities, including economic insecurity, living and working conditions, and social and community factors were present for YW pre-COVID but were exposed and amplified by the pandemic, resulting in a MH crisis for some YW. This is key, and needs to be addressed, as these adverse MH outcomes may have reached a critical threshold for young people in terms of their capacity to sustain the status quo, yet continue to fully participate in employment, given the post-COVID labour market and economic climate.

Despite YW’ challenging experiences during the pandemic, they demonstrated resilience and maintained an optimistic outlook. These strengths should be tapped into by government and employers, to both engage and empower Canadian YW, working collaboratively with them to address discrimination and improve their employment experiences and MH now and in the future. The recommendations of YW in this study address the compounding challenges they experienced during COVID-19 and provide meaningful policy-level solutions for structural changes, improved living and working conditions, and strengthened social and community support. Thus, it is critical that these recommendations be acted upon and implemented now to ensure stable and supportive futures for Canadian YW, enabling them to successfully share their knowledge and skills, safely participate in the workforce, and develop and support future Canadian economies.
