# Correction: The compounding impact of the social determinants of health and COVID-19 on the mental health of young workers in Canada during the COVID-19 pandemic: A qualitative, arts-based study

**DOI:** 10.1371/journal.pone.0320520

**Published:** 2025-03-17

**Authors:** Roberta L. Woodgate, Corinne A. Isaak, Julia Witt, Pauline Tennent, Ashley Bell

The captions for Figs 1 and 2 are incorrect. Please see the complete, correct captions of Figs 1 and 2 here.

**Fig 1:**
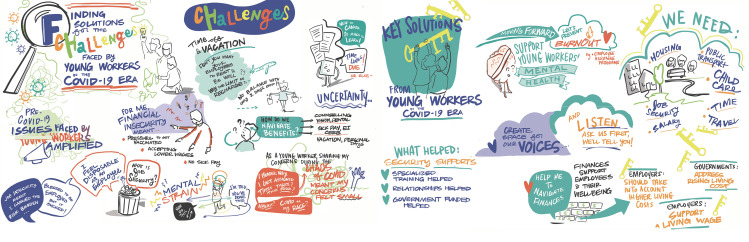
Graphic recording focus group.

**Fig 2:**
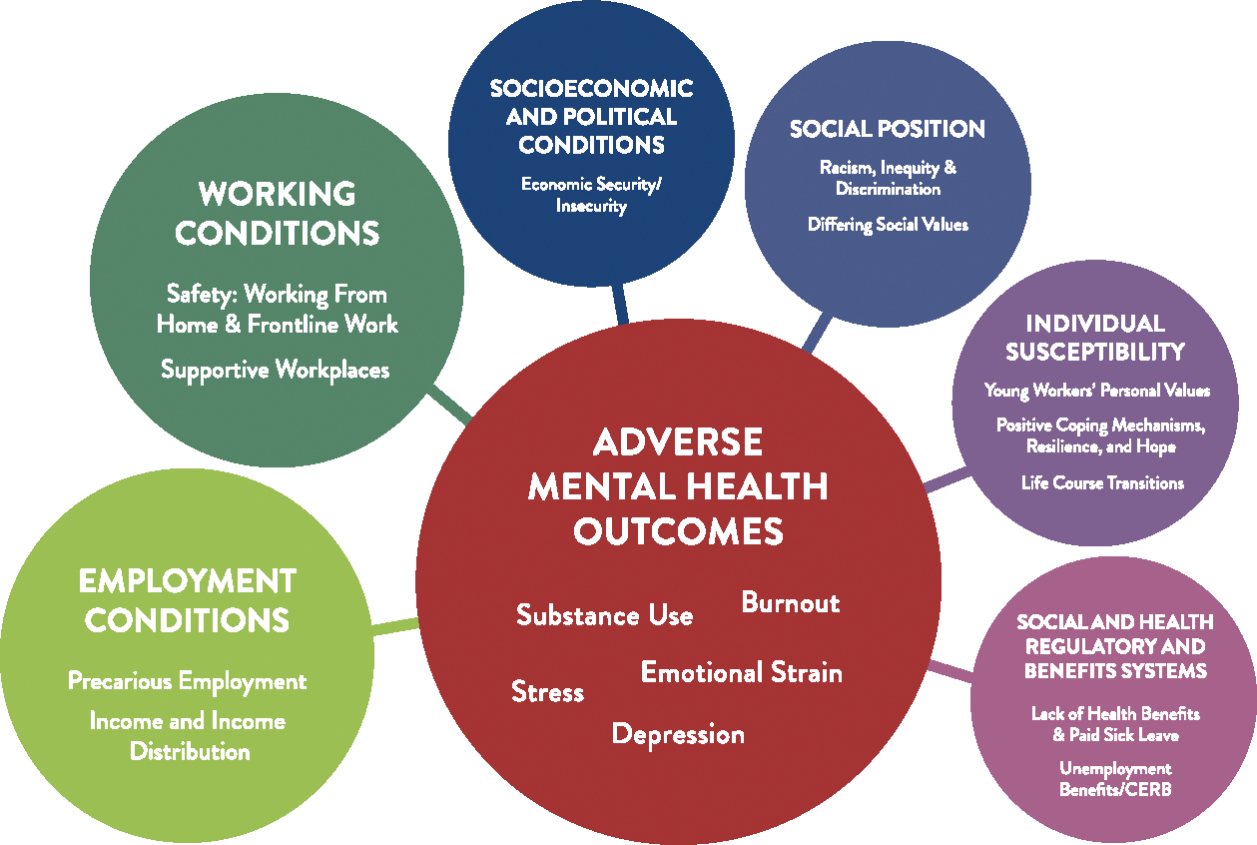
Compounding impact of the social determinants of health and covid-19 on young workers’ mental health.

In the Abstract section, there is an error in the 11^th^ sentence. The correct sentence is: Thus, it is critical that recommendations proposed by young workers in this study be acted upon and implemented to provide an equitable, stable, and supportive future for young workers in Canada and beyond.
